# Medical and sociodemographic characteristics related to feeding therapy referral and service provision for preterm infants in the neonatal intensive care unit

**DOI:** 10.1038/s41372-024-02184-y

**Published:** 2024-12-04

**Authors:** Tiana T. Nguyen, Roberta Pineda, Stacey Reynolds, Elizabeth E. Rogers, Audrey E. Kane

**Affiliations:** 1 Department of Physical Therapy and Rehabilitation Sciences, University of California, San Francisco, San Francisco, CA USA; 2 Department of Occupational Therapy, Samuel Merritt University, Oakland, CA USA; 3 Department of Occupational Therapy, Virginia Commonwealth University, Richmond, VA USA; 4 Chan Division of Occupational Science and Occupational Therapy, University of Southern California, Los Angeles, CA USA; 5 Division of Neonatology, Department of Pediatrics, University of California, San Francisco, San Francisco, CA USA

**Keywords:** Rehabilitation, Paediatrics

## Abstract

**Objective:**

To determine the scope of feeding therapy for preterm infants in the NICU and medical and sociodemographic factors related to feeding therapy referral and service provision.

**Study design:**

Retrospective study of infants born <37 weeks gestation in a level IV NICU between January 2017 and December 2019.

**Result:**

Among 547 infants, 27% of infants received a feeding therapy referral, and 74% of those referrals were problem-based referrals. Feeding therapy referrals were more likely among infants with lower gestational ages and birthweights (both *p* < 0.001). In addition, infants with greater medical complexity, who required oxygen at 36 weeks, who had a history of mechanical ventilation, and who had a higher postmenstrual age at discharge were more likely to be referred to feeding therapy (all *p* < 0.001).

**Conclusion:**

While medical factors relate to feeding therapy referrals, there are other complex person and system factors that determine feeding therapy referral and service provision.

## Introduction

Approximately 1 in 10 infants are born preterm (less than 37 weeks) in the United States [1]. When compared to their full-term counterparts, preterm infants have higher rates of disability, which may include feeding difficulties, cerebral palsy, developmental delay, vision problems, and hearing problems [1]. Social factors, such as maternal stress and low socioeconomic status, also increase the risk of developmental disabilities [2, 3]. Occupational therapy (OT), physical therapy (PT), and/or speech-language pathology (SLP) are rehabilitation services often recommended for preterm infants due to the high risk of developmental problems. A wide range of therapeutic interventions may be used to address the neurodevelopmental needs of preterm infants, with services often being initiated in the neonatal intensive care unit (NICU) [4].

In addition to general rehabilitation services, preterm infants often need expert support for feeding, preferably by feeding therapists with specialized knowledge and skills in infant feeding issues. Feeding therapists usually include OTs and SLPs; however, other disciplines may also be involved in helping infants and families attain successful feeding behaviors and practices. In the NICU setting, feeding therapists address swallowing issues and facilitate oral feeding development by providing interventions related to feeding and swallowing performance [4]. Feeding therapy interventions include oral motor exercises [5–7] and cue-based feeding [8–10], which have been found to be effective for the development of oral feeding skills for infants in the NICU. Other feeding therapy interventions such as positioning, pacing, adjusting the flow rate, thickening, and altering the temperature of the liquid are techniques feeding therapists may use to develop coordination to rhythmically suck-swallow-breathe and decrease the occurrence of liquid entering the airway [11–15].

Feeding therapy is an intervention approach often recommended for preterm infants in the NICU who present with, or at risk for, inadequate oral feeding [16, 17]. This is because prematurity and associated medical factors (e.g., immature lung development) can impact body systems, reflexes, and responses needed for successful oral feeding. Failure of an infant to achieve normal oral feeding milestones may delay NICU discharge, making it a priority of NICU care [18–21].

Despite evidence supporting early feeding therapy interventions for high-risk infants, there are no studies describing the utilization of feeding therapy for preterm infants in the NICU. Thus, we aimed to 1) determine the scope of feeding therapy for preterm infants in a level IV NICU, and 2) identify medical and sociodemographic factors related to feeding therapy referrals and service provision. We hypothesized that infants with greater medical complexity would be more likely to receive feeding therapy services in the NICU.

## Methods

### Study design

This study used a retrospective study design to analyze data from the electronic medical records (EMR) at the University of California, San Francisco (UCSF) Benioff Children’s Hospital NICU. Eligible participants were born at less than 37 weeks of gestation and were admitted to the study site NICU between January 2017 and December 2019. Preterm infants who had transferred from another hospital, transferred to another hospital, died, or born via surrogacy were excluded from the study. Infants with a long length of stay ( > 3 standard deviations from the mean; 114 days) were also excluded as this was believed to represent a different population of infants to study with extremely complicated medical courses. This study, including a waiver of informed consent, was reviewed and approved by the UCSF Institutional Review Board (#22-38263).

### Study site

UCSF Benioff Children’s Hospital has a 60-bed Level IV NICU that is associated with the UCSF Birth Center. Within its NICU, feeding therapists consist of OTs and SLPs with expertise in infant feeding and swallowing and who have completed the feeding and swallowing competency program at UCSF Benioff Children’s Hospital. OTs on this team must also obtain an advanced practice certification in swallowing assessment, evaluation, and intervention from the California Board of Occupational Therapy. Feeding and swallowing interventions used at this site are in alignment with what is reported in the literature and include addressing the development of the infant’s oral motor skills, using cue-based feeding techniques, positioning, pacing, adjusting flow rate, thickening, and altering the temperature of the liquid. OTs and SLPs share the caseload and there are usually 1 to 2 feeding therapists covering the NICU 5 days a week. On average, infants who are identified as needing feeding therapy services are seen twice a week for feeding therapy. Medical providers determined when the feeding therapy referral was placed based on their individual assessment of the infant’s identified medical risk. In this study, automatic referrals were defined as referrals from a medical provider that were placed prior to initiating oral feeding for the preterm infants, usually before 34 weeks postmenstrual age (PMA). Problem-based referrals were defined as referrals from a medical provider that were placed after the infant had started orally feeding, usually after 36 weeks PMA, and a feeding and/or swallowing concern arose that was related to either bottle or breastfeeding.

### Data extraction and cleaning

A clinical informaticist extracted the data from the EMR at UCSF and transferred de-identified data to a secure platform. There were a total of 1136 participants that met the inclusion and exclusion criteria. Descriptive statistics were used to check for normality, conduct data cleaning, and check for missing values. Outliers and missing values were checked for accuracy by the clinical informaticist who validated the data against the original source. Legitimate outliers were included in the analysis. If the value was truly missing, then that participant was eliminated by using complete case analysis [22]. The following variables were missing: 383 (33.3%) of birthweight, 298 (25.9%) of type of delivery, 193 (16.8%) of maternal age, 81 (7%) of infant race, 104 (9.1%) of ethnicity, and 196 (17.1%) of zip code, leaving a remainder of 547 participants. There were differences in history of mechanical ventilation, insurance, maternal marital status, and preferred language among those who had incomplete data and those who had complete data and were included in the study.

### Measures

*Feeding therapy service factors* were the primary outcomes of interest which included the receipt of a feeding therapy referral, type of referral (automatic or problem-based), and number of therapy sessions across the length of NICU stay.

*Sociodemographic factors* included infant’s sex assigned at birth, infant’s race and ethnicity as identified by the parent, preferred language, maternal age, maternal marital status, maternal mental illness, child protective services involvement during NICU stay, homelessness during NICU stay, infant’s insurance, maternal employment, maternal social support, and the distance the mother lived from the hospital. Distance was categorized based on the radius of the city of San Francisco (SF) ( < 7 miles), San Francisco Bay Area (7-47 miles), or outside of the SF Bay Area ( > 47 miles). Maternal mental illness included having a diagnosis of postpartum depression, substance abuse, and/or mood disturbance and was identified by the physician. Maternal social support was reported by the mother during the infant’s NICU stay, and was recorded as support from family, friends, and/or community members.

*Medical factors* included gestational age at birth, birthweight, type of delivery, medical complexity (as defined by the Pediatric Medical Complexity Algorithm) [23], requiring oxygen at 36 weeks PMA, history of mechanical ventilation, multiple birth, length of stay, and PMA at discharge. The Pediatric Medical Complexity Algorithm was used to classify children in the following categories of medical complexity: without chronic disease (acute non-chronic conditions or are healthy), with non-chronic complex disease (chronic condition in one body system), and with chronic complex disease (significant chronic conditions in at least two body systems or have a progressive condition) [23].

#### Data analysis

IBM Statistical Product and Service Solution 29.0 was used for data analysis. First, descriptive statistics were used to look at frequencies and central tendencies of descriptive factors as well as feeding therapy service factors. Then, Pearson’s chi-squared and independent samples *t*-tests were used to determine relationships between sociodemographic and medical factors and feeding therapy referral (received versus did not receive feeding therapy referral) as well as type of referral (automatic versus problem-based). Lastly, univariate linear regression was used to assess the relationships between sociodemographic and medical factors and the number of therapy sessions. Significance was set at 0.05. All variables that were identified as being independently predictive of the outcomes were also evaluated to determine if they were related to each other.

## Results

### Feeding therapy referral

A total of 547 preterm infants were included in the analysis and 150 (27%) of them received a feeding therapy referral (see Table 1). A majority of referrals were problem-based referrals (73%). Infants received automatic referrals at an average PMA of 33.0 weeks and problem-based referrals at an average PMA of 36.6 weeks.

**Table 1:** Number of infants who received feeding therapy referral, their median number of therapy sessions, and their mean post-menstrual age at receipt of feeding therapy referral.

	*N* (% of sample) (*N* = 547)	Median number of feeding therapy sessions (range)	Mean PMA at receipt of feeding therapy referral (SD)
Did not receive feeding therapy referral	397 (73%)		
Received feeding therapy referral	150 (27%)	3 (0–25)	35.5 (6.8)
Automatic referrals	39 (26%)	4 (0–25)	33.0 (3.8)
Problem-based referrals	111 (74%)	3 (0–25)	36.3 (7.3)

On average, infants included in this study were born at a gestational age of 33.6 weeks, with a birthweight of 2128.6 grams and length of stay of 21.7 days (see Table 2). The majority of infants had no complex chronic disease (93%), were White (37%), were English-speaking (94%), and had commercial insurance (62%).

**Table 2:** Participant characteristics related to receipt of feeding therapy referrals.

Participant characteristics	Overall *N* (%) or mean ± SD (*N* = 547)	Did not receive feeding therapy referral *N* (%) or mean ± SD (*n* = 397, 73%)	Received feeding therapy referral *N* (%) or mean ± SD (*n* = 150, 27%)	*p*-value
Infant sex^a^				0.074
Female	249 (46%)	190 (48%)	59 (39%)	
Male	298 (54%)	207 (52%)	91 (61%)	
Infant race and ethnicity^a^				0.082
White	205 (37%)	143 (36%)	62 (41%)	
Hispanic	109 (20%)	88 (22%)	21 (14%)	
Asian	92 (17%)	73 (19%)	19 (13%)	
Black	51 (9%)	33 (8%)	18 (12%)	
Other	49 (9%)	33 (8%)	16 (11%)	
Multiracial	41 (8%)	27 (7%)	14 (9%)	
Preferred language^a^				0.347
English	512 (94%)	374 (94%)	138 (92%)	
Not English	35 (6%)	23 (6%)	12 (8%)	
Maternal age^b^	38.0 ± 5.7	38.3 ± 5.8	37.3 ± 5.4	0.050*
Maternal marital status^a^				0.125
Married	356 (65%)	266 (67%)	90 (60%)	
Not married	191 (35%)	131 (33%)	60 (40%)	
Maternal mental illness^c^				
No	541 (99%)	393 (99%)	148 (99%)	
Yes	6 (1%)	4 (1%)	2 (1%)	
Child Protective Services involvement^c^				
No	544 (99%)	395 (99%)	149 (99%)	
Yes	3 (1%)	2 (1%)	1 (1%)	
Homelessness (current)^c^				
No	541 (99%)	396 (100%)	145 (97%)	
Yes	6 (1%)	1 (0%)	5 (3%)	
Infant insurance^a^				0.116
Commercial	339 (62%)	254 (64%)	85 (57%)	
Public (Medi-Cal)	208 (38%)	143 (36%)	65 (43%)	
Maternal employment^a^				<0.001*
Yes	255 (47%)	179 (45%)	76 (51%)	
No	118 (21%)	73 (18%)	45 (30%)	
Unknown	174 (32%)	145 (37%)	29 (19%)	
Maternal social support^a^				0.053
Yes	509 (93%)	368 (93%)	141 (94%)	
No	16 (3%)	9 (2%)	7 (5%)	
Unknown	22 (6%)	20 (5%)	2 (1%)	
Distance^a^				0.044*
<7 miles	221 (40%)	170 (43%)	51 (34%)	
7-47 miles	196 (36%)	143 (36%)	53 (35%)	
>47 miles	130 (24%)	84 (21%)	46 (31%)	
Gestational age at birth^b^	33.6 ± 2.3	34.0 ± 1.9	32.4 ± 2.9	<0.001*
Birthweight^b^	2128.6 ± 606.4	2232.0 ± 582.5	1854.8 ± 584.7	<0.001*
Type of delivery^a^				0.052
Vaginal	274 (50%)	209 (53%)	65 (43%)	
Caesarian section	273 (50%)	188 (47%)	85 (57%)	
Medical complexity^a^				<0.001*
Without complex chronic disease	508 (93%)	379 (96%)	129 (86%)	
With non-complex chronic disease	29 (5%)	13 (3%)	16 (11%)	
With chronic disease	10 (2%)	5 (1%)	5 (3%)	
Oxygen requirement at 36 weeks PMA^a^				<0.001*
No	489 (89%)	368 (93%)	121 (81%)	
Yes	58 (11%)	29 (7%)	29 (19%)	
History of mechanical ventilation^a^				<0.001*
No	501 (92%)	384 (97%)	117 (78%)	
Yes	46 (8%)	13 (3%)	33 (22%)	
Multiple birth^a^				0.503
No	405 (74%)	297 (75%)	108 (72%)	
Yes	142 (26%)	100 (25%)	42 (28%)	
Length of stay^b^	21.7 ± 21.0	15.4 ± 13.8	38.6 ± 26.8	<0.001*
PMA at discharge^b^	36.7 ± 1.6	36.2 ± 1.2	37.9 ± 2.0	<0.001*

The following variables were related to each other: gestational age and birthweight; and gestational age and length of stay.

^a^*p*-value from Pearson’s chi-square test.

^b^*p*-value from independent samples *t*-test.

^c^*p*-value not calculated given small representation across cells.

*p < 0.05.

While examining sociodemographic factors, we found that infants who received feeding therapy referrals were more likely to have mothers who were younger, unemployed, and lived more than 47 miles from the hospital (see Table 2). When examining medical factors, we found that infants who received feeding therapy referrals were more likely to be born at younger gestational ages and lower birthweights. Infants who had more medical complexity, required oxygen at 36 weeks PMA, had a history of mechanical ventilation, had a longer length of stay, and had a higher PMA at discharge were more likely to receive feeding therapy referrals.

### Type of feeding therapy referral

Infants who received an automatic referral were more likely to be born at younger gestational ages and lower birthweights (see Table 3). Infants who required oxygen at 36 weeks PMA, had a history of mechanical ventilation, had a longer length of stay, and had a higher PMA at discharge were more likely to have an automatic referral versus a problem-based referral. Although each of these variables was independently predictive of receipt of feeding therapies, many of these variables were related to one another, demonstrating the highly complex entanglement of different characteristics within the NICU population.

**Table 3:** Participant characteristics for infants who received automatic compared to problem-based referrals.

Participant characteristics	Automatic referrals *N* (%) or mean ± SD (*n* = 39, 26%)	Problem-based referrals *N* (%) or mean ± SD (*n* = 111, 74%)	*p*-value
Infant sex^a^			0.527
Female	17 (44%)	44 (38%)	
Male	22 (56%)	69 (62%)	
Infant race and ethnicity^b^			
White	27 (44%)	45 (40%)	
Hispanic	7 (18%)	14 (13%)	
Asian	6 (15%)	13 (12%)	
Black	4 (10%)	14 (13%)	
Other	3 (8%)	13 (12%)	
Multiracial	2 (5%)	12 (10%)	
Preferred language^a^			0.546
English	35 (90%)	103 (93%)	
Not English	4 (10%)	8 (7%)	
Maternal age^c^	38.2 ± 4.5	36.9 ± 5.6	0.221
Maternal marital status^a^			0.323
Married	26 (67%)	64 (58%)	
Not married	13 (33%)	47 (42%)	
Maternal mental illness^b^			
No	38 (97%)	110 (99%)	
Yes	1 (3%)	1 (1%)	
Child Protective Services involvement^b^			
No	39 (100%)	110 (99%)	
Yes	0 (0%)	1 (1%)	
Homelessness (current)^b^			
No	39 (100%)	106 (95%)	
Yes	0 (0%)	5 (5%)	
Infant insurance^a^			0.970
Commercial	22 (56%)	63 (57%)	
Public (Medi-Cal)	17 (44%)	48 (43%)	
Maternal employment^a^			0.967
Yes	20 (51%)	56 (50%)	
No	12 (31%)	33 (30%)	
Unknown	7 (18%)	22 (20%)	
Maternal social support^b^			
Yes	37 (95%)	104 (94%)	
No	2 (5%)	5 (4%)	
Unknown	0 (0%)	2 (2%)	
Distance^a^			0.708
<7 miles	12 (31%)	39 (35%)	
7-47 miles	13 (33%)	40 (36%)	
>47 miles	14 (36%)	32 (29%)	
Gestational age at birth^c^	30.2 ± 3.3	33.1 ± 2.4	<0.001*
Birthweight^c^	1475.0 ± 656.2	1988.2 ± 495.2	<0.001*
Type of delivery^a^			0.066
Vaginal	12 (31%)	53 (48%)	
Cesarean section	27 (69%)	58 (52%)	
Medical complexity^b^			
Without complex chronic disease	29 (74%)	100 (90%)	
With non-complex chronic disease	6 (16%)	10 (9%)	
With chronic disease	4 (10%)	1 (1%)	
Oxygen requirement at 36 weeks PMA^a^			0.010*
No	26 (67%)	95 (86%)	
Yes	13 (33%)	16 (14%)	
History of mechanical ventilation^a^			<0.001*
No	23 (59%)	94 (84%)	
Yes	16 (41%)	17 (15%)	
Multiple birth^a^			0.226
No	31 (79%)	77 (69%)	
Yes	8 (21%)	34 (31%)	
Length of stay^c^	60.5 ± 26.6	30.9 ± 22.4	<0.001*
PMA at discharge^c^	38.8 ± 2.3	37.5 ± 2.3	<0.001*

Gestational age, birthweight, and length of stay were related to each other.

^a^*p*-value from Pearson’s chi-square test.

^b^*p*-value not calculated given small representation across cells.

^c^*p*-value from independent samples *t*-test.

*p < 0.05.

### Number of feeding therapy sessions

Infants whose parents had identified them as Hispanic, Black, other, or multiracial received more feeding therapy sessions than infants identified as White, while infants identified as Asian received less feeding therapy sessions than infants identified as White (see Table 4). Infants who had public insurance (Medi-Cal) received more feeding therapy sessions than infants who had private insurance. Infants with lower gestational age at birth, higher medical complexity, oxygen requirement at 36 weeks PMA, history of mechanical ventilation, longer length of stay, and higher PMA at discharge received more feeding therapy sessions.

**Table 4:** Univariate linear regression of associations between participant characteristics and number of feeding therapy sessions.

Participant characteristics	Unstandardized beta (S.E.)	95% CI		*p*-value
		Lower	Upper	
Infant sex				
Female	Ref			
Male	0.207 (0.763)	−1.300	1.715	0.786
Infant race and ethnicity				0.009*
White	Ref			
Hispanic	4.016 (1.108)	1.825	6.207	
Asian	−0.352 (1.151)	−2.628	1.923	
Black	1.016 (1.175)	−1.307	3.341	
Other	2.079 (1.231)	−0.355	4.512	
Multiracial	1.373 (1.299)	−1.194	3.941	
Preferred language				0.345
English	Ref			
Not English	1.297 (1.370)	−1.410	4.004	
Maternal age	−0.057 (0.069)	−0.195	0.080	0.411
Maternal marital status				0.234
Married	Ref			
Not married	0.906 (0.757)	−5.91	2.402	
Infant insurance				0.023*
Commercial	Ref			
Public (Medi-Cal)	1.703 (0.739)	0.243	3.164	
Maternal employment				0.205
Yes	Ref			
No	1.099 (0.852)	−0.585	2.784	
Unknown	−0.755 (0.989)	−2.709	1.199	
Maternal social support				0.836
Yes	Ref			
No	0.914 (1.771)	−2.587	4.415	
Unknown	−0.943 (3.258)	−7.381	5.496	
Distance				0.223
<7 miles	Ref			
7-47 miles	−0.948 (0.889)	−2.705	0.810	
>47 miles	0.621 (0.922)	−1.200	2.443	
Gestational age at birth	−0.320 (0.125)	−0.567	−0.073	0.011*
Birthweight	−0.001 (0.001)	−0.002	0.000	0.175
Type of delivery				
Vaginal	Ref			
C-section	0.387 (0.751)	−1.098	1.872	0.607
Medical complexity				0.002*
Without complex chronic disease	Ref			
With non-complex chronic disease	2.160 (1.163)	−0.138	4.458	
With chronic disease	6.473 (2.000)	2.521	10.425	
Oxygen requirement at 36 weeks PMA^1^				0.008*
No	Ref			
Yes	2.470 (0.922)	0.648	4.291	
History of mechanical ventilation				0.042*
No	Ref			
Yes	1.821 (0.887)	0.068	3.574	
Multiple birth				0.177
No	Ref			
Yes	−1.120 (0.825)	−2.751	0.510	
Length of stay	0.063 (0.013)	0.038	0.089	<0.001*
PMA at discharge	0.919 (0.169)	0.586	1.253	<0.001*

Gestational age and length of stay were related to each other.

Ref = reference group. Maternal mental illness, child protective services involvement, and homelessness were not included in this analysis due to small representation across cells.

*p < 0.05.

## Discussion

The purpose of this study was to determine patterns of feeding therapy referral for preterm infants in a level IV NICU and identify infant characteristics associated with feeding therapy referral and service provision. Over a fourth (27%) of preterm infants in the UCSF NICU received a feeding therapy referral, with medically complex infants being more likely to be referred. The majority of referrals (74%) were problem-based referrals, with automatic referrals more likely among infants who ended up having longer NICU stay. There was variability in the number of therapy sessions provided to infants after the initial referral, ranging from zero to 25. Both social and medical factors were found to be associated with the number of therapy sessions received. These findings suggest that there is an opportunity to optimize feeding therapy referrals in the NICU and increase service uptake by developing a more consistent approach to delivering therapy services for infants at risk of feeding difficulties.

This study found that medical factors (gestational age, birthweight, medical complexity, oxygen requirement at 36 weeks PMA, history of mechanical ventilation, length of stay, PMA at discharge) were associated with receipt of feeding therapy referral, referral type, and number of therapy sessions received. Medical providers mainly appeared to be ordering feeding therapy referrals based on identified feeding problems (problem-based referrals) that could be related to either bottle or breastfeeding. Referrals for feeding therapy were made for a wide range of problems that include, but not limited to, poor feeding readiness, inefficient oral feeding pattern, poor coordination of suck-swallow-breathe, poor progression of oral feeding, or aspiration concerns (e.g., coughing, choking, physiological instability). Although automatic referrals for feeding therapy were used less frequently, they were used more often with infants who were anticipated to be hospitalized longer and in groups of infants with high risk of feeding challenges [17]. Automatic referrals were placed on average at 33 weeks PMA and infants begin to coordinate sucking and swallowing around 32 to 34 weeks [24]. Our findings suggest that feeding therapy can be initiated at an early PMA prior to initiating oral feeding to support oral motor skill development that is necessary for oral feeding success.

Similar to Butera et al. and Ross et al., this study found that therapists can provide early therapeutic interventions for medically complex infants within the NICU and that infants who are at higher risk for developmental delays have more therapy sessions [4, 25]. Infants who received more therapy sessions were more likely to have complex medical conditions. Infants who are more medically complex likely have longer length of stays, and infants who are hospitalized for longer inherently have more time to receive therapy sessions. Further, infants with a history of mechanical ventilation and who continue to require oxygen at 36 weeks were more likely to receive a feeding therapy referral, for the physician to have exercised automatic referral, as well as to receive more feeding therapy sessions during their NICU stay. Respiratory status is closely linked to successful neonatal feeding, as infants must coordinate sucking, swallowing, and breathing [26] which could explain the link between the presences of respiratory conditions (history of mechanical ventilation and requiring oxygen support at 36 weeks) and feeding therapy referral and number of therapy sessions. As an institution, it is not our practice to orally feed preterm infants while on continuous positive airway pressure support. Rather the focus of feeding therapy is on oral motor and sensory development until the infant is on lower levels of respiratory support to coordinate suck, swallow, and breathing necessary for oral feeding.

In addition to factors related to long hospitalization and risk of feeding challenges, infants who had families that lived further away, mothers that were employed, or younger mothers were more likely to receive a feeding therapy referral. It is possible that families who lived outside of the San Francisco Bay Area specifically came to UCSF, as it is one of the regional Level IV NICUs, to give birth if they were experiencing a high-risk pregnancy. Providers may be more inclined to order therapy services for families who lived further away, as these families may not have been able to visit as easily as families who lived closer. Similarly, if the mother was employed and unable to routinely visit and feed their infant in the NICU, it is possible that providers sensed more of a need for therapy intervention. Lastly, providers may feel that younger mothers may benefit from more education that feeding therapists could provide, especially if this is their first child. More research needs to be done to determine the cause of these relationships.

This study identified relationships between race and socioeconomic status and feeding therapy referral and number of therapy sessions in the NICU. Infants who were Hispanic or had public insurance (Medi-Cal) received more feeding therapy sessions. Infants who identified as Black, Hispanic, other, and multiracial had more therapy sessions than infants who identified as White. Our findings are consistent with Butera et al. who also found that White infants received less therapy sessions than non-White infants within a NICU setting [25]. Infants who also had public insurance (Medi-Cal) had a higher number of feeding therapy sessions than those who have private insurance. In contrast, previous literature identified higher intensity of occupational therapy services among individuals that have private insurance [27, 28]. The differences may be due to practice setting and populations served. Previous research has identified that those with public insurance or who are uninsured have poorer maternal and neonatal outcomes [29, 30]. Thus, if infants with public insurance have more medical risk, then it is possible that they were provided with more therapy sessions.

We did not observe associations between the other social risk factors, such as family structure or primary language, and feeding therapy referral or number of therapy sessions, despite other studies reporting disparities in therapy service delivery after NICU discharge [31]. This may be due to the difference in barriers associated with hospital-based versus community-based care. When a referral is placed within the community, it is possible that barriers, such as scheduling, transportation, and family follow-through, may prevent infants from receiving therapy services. Within the hospital setting, these barriers may not impede receipt of services for infants in the NICU.

To our knowledge, this is the first study that describes patterns of feeding therapy referral and service provision in a level IV NICU. The results from this study contribute to our understanding of how referrals and feeding therapy uptake occur in the NICU based on infant characteristics. Understanding who gets feeding therapy and how feeding therapy is utilized is the first step towards improving service delivery and subsequently, feeding outcomes. The results highlight the potential for feeding therapists to be more effectively integrated into the NICU team to address the unique developmental and feeding needs of preterm infants, thereby mitigating the neurodevelopmental sequelae of prematurity. Future research is needed to determine the optimal timing and frequency of feeding therapy services to achieve the best developmental and feeding outcomes.

### Limitations

Findings from this study may not generalize to all NICU settings. There can be variability in the role feeding therapists play on the NICU team based on organizational and individual factors. Not all hospitals have feeding therapists in the NICU, and there is significant variability across settings in terms of the role of feeding therapy based on factors like therapist availability, the population(s) that they serve, training, and experience. The feeding therapy team in the NICU could also consist of different professions, such as OT, physical therapy, and/or SLP. Future studies should include varying levels of NICUs in different geographical locations expanding upon our findings and to enhance external validity.

Another limitation that should be acknowledged is that reliability of retrospective data is impacted by how therapists and medical providers recorded the information in the EMR. There were many participants excluded due to incomplete data. Staffing and census data could not be collected through the EMR for this study. Decreased staffing or high census could impact the number of feeding therapy sessions provided, but this information was not available. Direct caregiver involvement in therapy sessions was also not collected. This could also impact the number of therapy sessions provided. Finally, the type and quality of the therapy sessions were not ascertained from the EMR documentation. Future studies could also include feeding outcomes to determine the effectiveness of and timing of feeding therapy.

## Data Availability

The data used to support the findings of this study is available from the corresponding author upon reasonable request.
